# Direct Evidence of the Exfoliation Efficiency and Graphene Dispersibility of Green Solvents toward Sustainable Graphene Production

**DOI:** 10.1021/acssuschemeng.2c03594

**Published:** 2022-12-09

**Authors:** Kai Ling Ng, Barbara M Maciejewska, Ling Qin, Colin Johnston, Jesus Barrio, Maria-Magdalena Titirici, Iakovos Tzanakis, Dmitry G Eskin, Kyriakos Porfyrakis, Jiawei Mi, Nicole Grobert

**Affiliations:** † Department of Materials, University of Oxford, Parks Road, OxfordOX1 3 PH, U.K.; ‡ Department of Engineering, University of Hull, Cottingham Road, HullHU6 7RX, U.K.; § Department of Chemical Engineering, Imperial College London, South Kensington Campus, LondonSW7 2AZ, U.K.; ∥ School of Engineering, Computing and Mathematics, Oxford Brookes University, College Cl, Wheatley, OxfordOX33 1HX, U.K.; ⊥ Brunel Centre for Advanced Solidification Technology, Brunel University London, Kingston Lane, UxbridgeUB8 3PH, U.K.; # Faculty of Engineering and Science, University of Greenwich, Central Avenue, Chatham Maritime, KentME4 4TB, U.K.; ¶ Williams Advanced Engineering, Grove, OxfordshireOX12 0DQ, U.K.

**Keywords:** graphene, liquid phase exfoliation, green solvents, NMP, exfoliation efficiency, dispersibility, re-dispersion

## Abstract

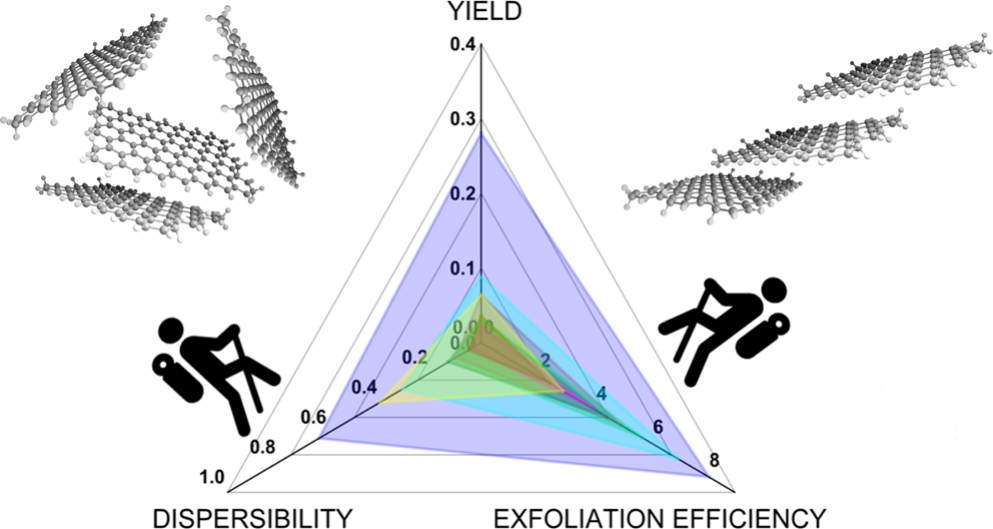

Achieving a sustainable production of pristine high-quality graphene and other layered materials at a low cost is one of the bottlenecks that needs to be overcome for reaching 2D material applications at a large scale. Liquid phase exfoliation in conjunction with *N*-methyl-2-pyrrolidone (NMP) is recognized as the most efficient method for both the exfoliation and dispersion of graphene. Unfortunately, NMP is neither sustainable nor suitable for up-scaling production due to its adverse impact on the environment. Here, we show the real potential of green solvents by revealing the independent contributions of their exfoliation efficiency and graphene dispersibility to the graphene yield. By experimentally separating these two factors, we demonstrate that the exfoliation efficiency of a given solvent is independent of its dispersibility. Our studies revealed that isopropanol can be used to exfoliate graphite as efficiently as NMP. Our finding is corroborated by the matching ratio between the polar and dispersive energies of graphite and that of the solvent surface tension. This direct evidence of exfoliation efficiency and dispersibility of solvents paves the way to developing a deeper understanding of the real potential of sustainable graphene manufacturing at a large scale.

## Abstract 2

Deconvolution of the overall graphene yield of green solvents into exfoliation efficiency and dispersibility. This deconvolution reveals the high exfoliation efficiency of green solvents.

## Introduction

Graphene is widely investigated as the next generation material for application in flexible electronics,^1^ electrocatalysts,^2^ bioscaffolds,^3^ sensors,^4^ etc. For such applications, graphene is generally produced by simple, scalable, and inexpensive liquid phase exfoliation (LPE) techniques whereby graphite crystals are either shear-mixed (SM) or ultrasonically exfoliated in a solvent. In LPE, there are three key factors that influence and govern the graphene yield: the (i) graphite quality, the (ii) exfoliation efficiency, and the (iii) dispersibility of the solvent. The quality of graphite, defined by the graphite size, crystallinity, and impurities, greatly affects its wettability.^5−7^ The higher the graphite quality, the lower its wettability, and if a solvent cannot wet the graphite, it cannot penetrate between the graphite layers to peel them off. Intuitively, this means that large, highly crystalline graphite flakes are more difficult to exfoliate than smaller, less crystalline, and defect-containing graphite powders. The exfoliation efficiency is defined by the ability of the solvent to “peel off” individual layers of graphene from the graphite crystal. In contrast to this, the graphene dispersibility is the ability of the solvent to form uniform and stable graphene dispersions over a given time. Typically, 1-methyl-2-pyrrolidone (NMP) or dimethylformamide (DMF) is used as a solvent in LPE since both are ideal for exfoliating graphite and producing stable dispersions of graphene.^8^ However, their toxicity and high boiling points, for example, 153 and 202 °C for DMF and NMP, respectively, are an issue in the context of sustainable graphene manufacturing, and therefore, alternative LPE solutions must be sought out.^9−14^ High-boiling-point solvents are difficult to remove and result in major loss of the material, degradation of the material quality, and the generation of toxic waste.^13,15^ Therefore, major significant efforts have gone into finding suitable and sustainable solvent alternatives, so-called green solvents.^13,16^ Green solvents are eco-friendly solvents with low boiling points, for example, <100 °C, and low toxicity.^17^ Although previous work has shown that green solvents can indeed be used to produce graphene by means of LPE, it has not progressed much further. To enhance the graphene yield in green solvent LPE (GS-LPE), which currently is ca. 50–75% lower than in DMF or NMP,^18,19^additives such as surfactants and/or dispersants (sodium cholate, sodium dodecylsulfate, Pluronic, etc.) have been intensively explored.^20−23^ Unfortunately, additives remain on the graphene and are difficult to remove after exfoliation, and therefore, the as-produced graphene is no longer pristine, which can affect its properties and negatively influence its performance.^24^ In the pursuit of improving the graphene yield of the GS-LPE process, solvent exchange methods have also been investigated, whereby graphite was first “pre-treated” and/or exfoliated in NMP or DMF, followed by re-dispersion of the as-produced graphene in a green solvent.^25−27^ While the graphene yield could indeed be increased through the solvent exchange, toxic NMP and DMF solvents are still required for the exfoliation. This will contribute in developing an understanding of the actual role that the solvent plays in the LPE mechanism and how it specifically contributes toward the exfoliation process. The current evaluation method of the graphene yield in green solvents is hindered by the limited dispersibility of the as-produced graphene in green solvents. The low amount of collected (and detected) graphene gives the false impression that green solvents are unsuitable for the efficient production of graphene. This study paves the way for the development of a set of post-exfoliation procedures for the enhancement of the graphene yield in green solvents through the optimization of their dispersibility. This route opens up new opportunities to explore the real potential of green solvents, thought of as inefficient in the production of graphene via LPE.

## Results and Discussion

**Figure 1:**
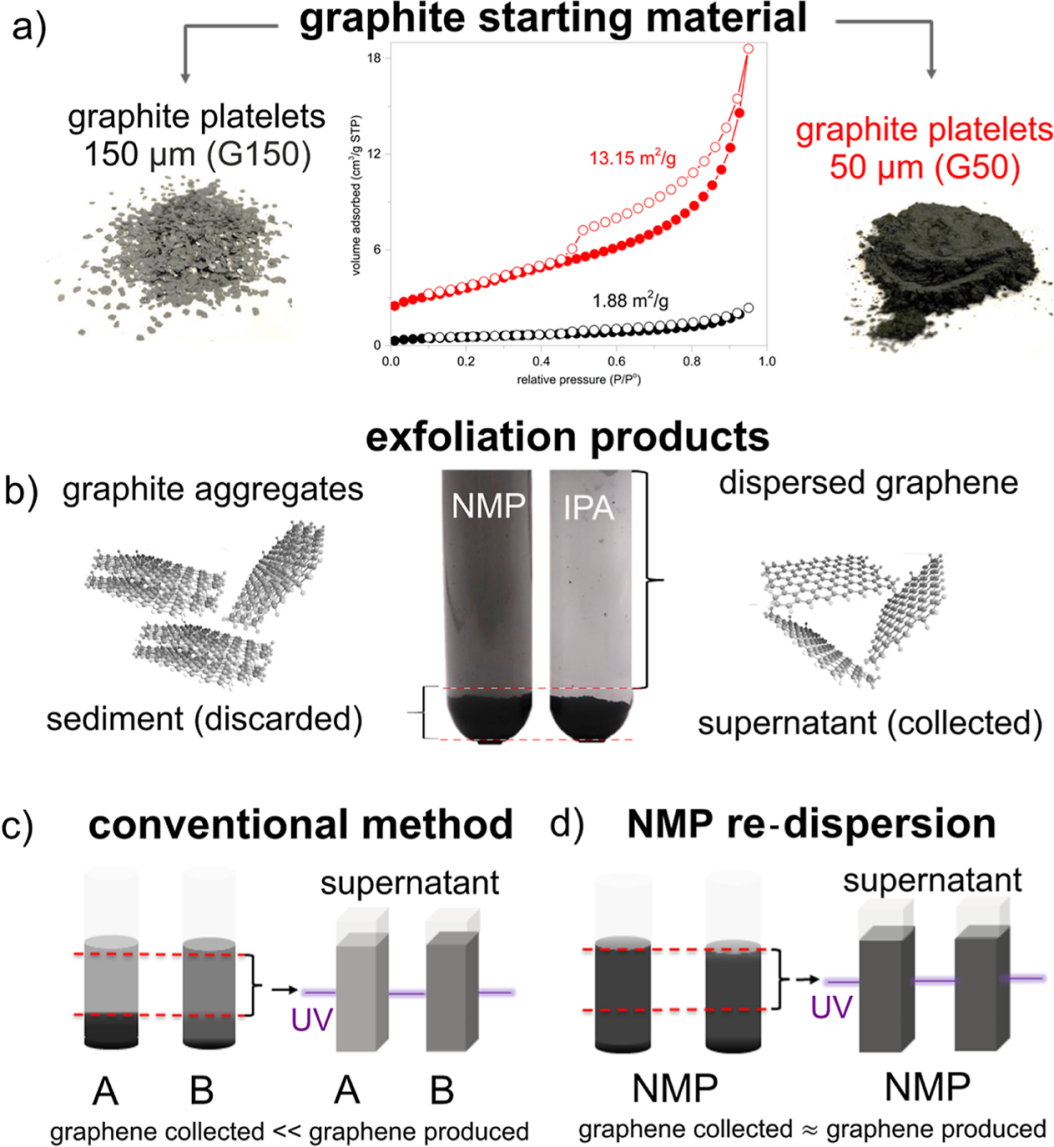
(a) Digital images of graphite platelets (150 μm; G150) and powder (50 μm; G50); BET surface area evaluation of both. (b) Centrifuge tubes with a fixed amount of exfoliation product containing graphite aggregates and graphene dispersed in NMP and IPA, respectively. When the exfoliation product contains the same amount of graphene, solvents with a higher graphene dispersibility result in a higher concentration of graphene collected from the supernatant. (c) For the conventional method, the concentration characterized is limited by the graphene dispersibility in the solvent. Due to the lower graphene dispersibility in solvent A, the exfoliated graphene restacks and sediments during centrifugation. This results in a lower amount of graphene being collected from the supernatant, and thus, a lower concentration is measured, despite the same initial amount of graphene content as in solvent B. (d) The NMP-R method makes use of the higher graphene dispersibility of NMP to minimize graphene restacking so that the graphene produced remains well-separated in the supernatant. Note that the black and red graphs depict the data for G150/GR150 and G50/GR50, respectively.

To study the feasibility of replacing NMP and DMF with green solvents (S1.1), we employed shear mixing LPE (SM-LPE, S1.2) in conjunction with distinctively different graphite materials (Figure 1a), that is, graphite platelets (G150) and graphite powder (G50) with lateral sizes of 150 and 50 μm, respectively (Figure S1), and their surface areas of 13.15 (G50) and 1.88 m^2^/g (G150) were measured by the Brunauer–Emmet–Teller (BET) N_2_ absorption isotherms. The experimental methods used for the exfoliation and re-dispersion of the exfoliated graphene are detailed in S1.2. The characterization techniques used for the graphite and graphene materials are detailed in S2. Following the work by Coleman et al., it is generally accepted that a higher graphene yield can be achieved if the surface tension or solubility parameters of a solvent are similar to that of graphene.^11,28^ This rule-of-thumb is typically employed to select the solvents for the LPE of layered materials.^29^ The surface energy of ideal graphene, that is, atomically perfect graphene, is 68 mJ/m^2^.^29,30^ This value changes for defective graphene and may vary for graphite due to, for example, graphite morphology, lateral size, defect density, edge defects, *d*-spacing, additional functional groups, and so forth.^5,31^ We deliberately selected graphite starting materials with different degrees of defect in our study (S3). G50 graphite is more defective than G150, with a higher D peak at (1350 cm^–1^ Raman shift), as analyzed through Raman spectroscopy (Figure S1). Herein, we employed SM-LPE in a range of green solvents [acetone (Ace), methanol (MeOH), ethanol (EtOH), isopropanol (IPA), ethyl acetate (EA), IPA, and (1:1) volume ratio of EtOH/deionized (D.I.) water and IPA/Ace] while keeping shear mixing parameters constant (S1.2.1). As the shear mixing product usually consists of well-separated/-dispersed graphene and graphite aggregates composed of stacked graphene and partially exfoliated graphite (Figure 1b), centrifugation is employed to separate the graphene-rich phase (supernatant) from other exfoliation products. If the solvent dispersibility is poor, however, as in the case for graphene in green solvents, the exfoliated graphene starts to restack and sediments during centrifugation. In such a case, the graphene collected in the supernatant phase is much lower than the actual amount of graphene originally exfoliated, and the graphene concentration is limited to the amount that a given green solvent can disperse. For example, if the same amount of graphene is dispersed in two solvents (A and B) with different graphene dispersibilities, where solvent B has a higher dispersibility, the concentration of graphene measured from the supernatant solvent B would be higher than that of solvent A (Figure 1c) and therefore would distort the measurement of the actual graphene concentration.

**Figure 2:**
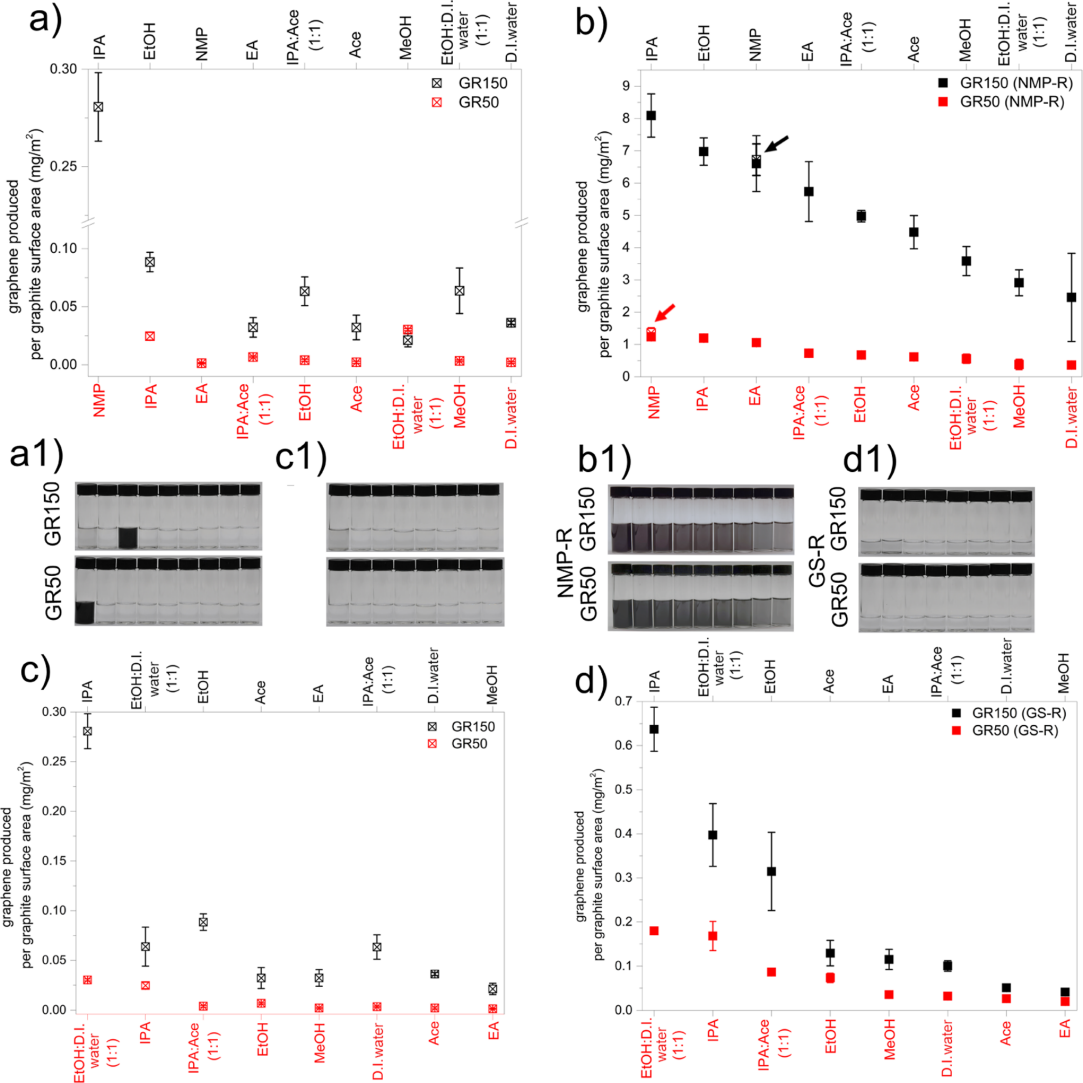
Mass of GR150 and GR50 graphene produced per surface area of G150 and G50 graphite, respectively, exfoliated in various exfoliation solvent media (*x*-axis), determined through the conventional method (without re-dispersion), NMP-R, and GS-R. (a) Conventional method in comparison with the (b) NMP-R. The solvents on the *x*-axes of the graphs (a,b) are arranged in the order of decreasing solvent exfoliation efficiency. The data for NMP-exfoliated graphene in (a) is shown in (b) instead to compare with the NMP-R of the NMP-exfoliated graphene. (c) Conventional method in comparison with the (d) GS-R. The solvents on the *x*-axes of the graphs (c,d) are arranged in the order of decreasing dispersibility. (a1–d1) Digital images of graphene dispersion presented in (a–d), respectively, and arranged according to the solvent sequence in the *x*-axis of the graphs. The NMP-R graphene dispersions shown in (b1) are diluted five times for better contrast.

To study the green solvent exfoliation efficiency and dispersibility of graphene independently, we performed SM-LPE in a range of green solvents and then removed the solvent and re-dispersed the subsequent powder in NMP (with high dispersibility) for analysis only, referred to as the NMP re-dispersion (NMP-R) from hereon. Here, NMP merely serves as a standard dispersing medium, that is, tool, to investigate the exfoliation efficiency and dispersibility, independently. With this approach, it is possible to compare the actual yield of graphene in green solvents (Figure 1d). For the study of the green solvent dispersibility however, an inverse approach was taken, namely, the green solvent re-dispersion (GS-R). For the GS-R, graphite was first exfoliated in NMP; next, the LPE product was washed and dried and then re-dispersed in green solvents. In both cases, a vortex mixer was used for the re-dispersion, that is, no ultrasound or shear mixing was applied. The detailed experimental procedures of NMP-R and GS-R are presented in S1.2.2. Both NMP-R and GS-R routes can be applied to any graphite or other layered materials in order to identify the most efficient green solvent for the synthesis of these materials. UV–vis absorption spectra are the most frequently used for the evaluation of the actual graphene concentration and consequently graphene yield. In order to estimate the graphene concentration, the absorptivity value of graphene in a given solvent is required. It is a common practice to use the absorptivity values generated from calibration using only a single solvent medium, which is then used for the concentration calculation in various solvent media. In the literature, the type of solvent medium used for making a calibration curve for absorptivity value is often not specified.^11,16,29,32^ Here, we show the significance of selecting the right dispersing solvent medium for the calibration curve to generate a reliable standardized absorptivity value for the concentration calculation that can be applied to different dispersing solvent media used (S4). We compared the absorptivity values for GR150 and GR50 in both NMP and D.I. water (see Figure S2). Absorptivity values, ε, for GR150 and GR50 in NMP are used for the concentration calculation based on the Lambert–Beer law due to the high graphene dispersibility of NMP. ε for GR150 and GR50 are 1178 and 1589 mL mg^–1^ m^–1^, respectively. In most cases, the graphene yield or exfoliation efficiency is correlated with the initial weight of graphite used for LPE, and it is generally accepted that the graphite source is represented by its mass,^33^ while the graphite surface area is neglected or not considered to be important. For example, Lund et al. claimed that the concentration of graphene produced using LPE is independent on the size of the graphite source.^34^ Here, we demonstrate that the nature of the starting graphite material matters because the exfoliation efficiency and dispersibility are greatly affected by the crystallinity (e.g., *d*-spacing) and the size of the graphite crystal. Both the conventional GS-LPE (Figure 2a) and the GS-LPE combined with the NMP-R approach (Figure 2b) showed that the estimated mass per surface area for GR50 is much lower than that of GR150. This is most likely due to the much larger lateral size of G150 exposed to the direction parallel to the shear force generated between the rotor and the stator during LPE. This indicates the importance of considering the quality and surface area of the graphite starting materials in the evaluation of the LPE process capability to exfoliate graphene. Here, we normalized the graphene concentration and divided the mass of the exfoliated graphene by the surface area of the initial graphite source (Figure 2) in order to consider the structural difference between the types of graphite used (see also Figures S3 and S4 that showed the conventional method of concentration analysis without surface area normalization).

In conventional GS-LPE, the poor graphene dispersibility of the green solvents (besides NMP) results in a considerably lower graphene concentration in the supernatant after centrifugation than the actual amount of graphene produced (Figure 2a,a1); this is mainly because of the inability of the green solvent to hold the graphene in the suspension. To overcome the issue of poor solvent dispersibility in assessing the actual potential or exfoliation efficiency of a green solvent for LPE, we employed a solvent exchange method enabling the deconvolution of exfoliation efficiency and solvent dispersibility, both contributing to the yield, that is, the actual amount of graphene produced (Figure 2b1). For the NMP-R procedure, the graphene-rich exfoliation product, that is, the material that is collected after exfoliation but before centrifugation, is then re-dispersed in NMP (Figure 2b1). The NMP-R revealed up to 29 times’ higher mass of graphene per surface area for IPA-exfoliated GR150 and 48 times for IPA-exfoliated GR50. We re-dispersed the NMP-exfoliated GR150- and GR50-containing product back into the NMP, confirming that the concentration remained the same after NMP-R (Figure 2b, data marked with red and black arrows). This shows that NMP-R does not further exfoliate the material; it merely helped to disperse the exfoliated graphene. In this work, we show clear evidence for IPA possessing the highest exfoliation efficiency among the green solvents for both GR150 and GR50 (Figure 2a,b) (see also Figure S5a). Interestingly, the exfoliation efficiencies of both, IPA and EtOH, exceed that of NMP for GR150, whereas for GR50, the exfoliation efficiencies of IPA and EA are comparable to that of NMP (Figure 2b,b1). The evaluation of the contribution of the dispersibility of the solvent toward the actual yield was performed by the GS-R method (Figure 2c,d) (see also Figure S5b), in which G150 and G50 were first shear-mixed in NMP. The graphene-containing suspension was then filtered and washed with EtOH in order to remove any NMP residues, dried, and re-dispersed in selected green solvents. This evaluation explicitly shows that IPA has the best graphene dispersibility for a green solvent for GR150, followed by the EtOH/D.I. water, whereas for GR50, EtOH/D.I. water possesses better dispersibility than IPA (Figure 2d,d1). It is also evident that EA is a relatively good solvent to disperse GR150 but not GR50 (Figure 2d,d1), implying the dissimilar potential of a given solvent toward different graphite types (sources) used in GS-LPE. In contrast, D.I. water, Ace, and MeOH are generally recognized as poor solvents for exfoliation and dispersion of both, GR150 and GR50. The UV–vis spectra of GR150 and GR50 in different solvents are shown in Figure S5a,b. UV–vis spectra of NMP-exfoliated GR150 and GR50 re-dispersed in different green solvents (GS-R) are shown in Figure S5c,d.

**Figure 3:**
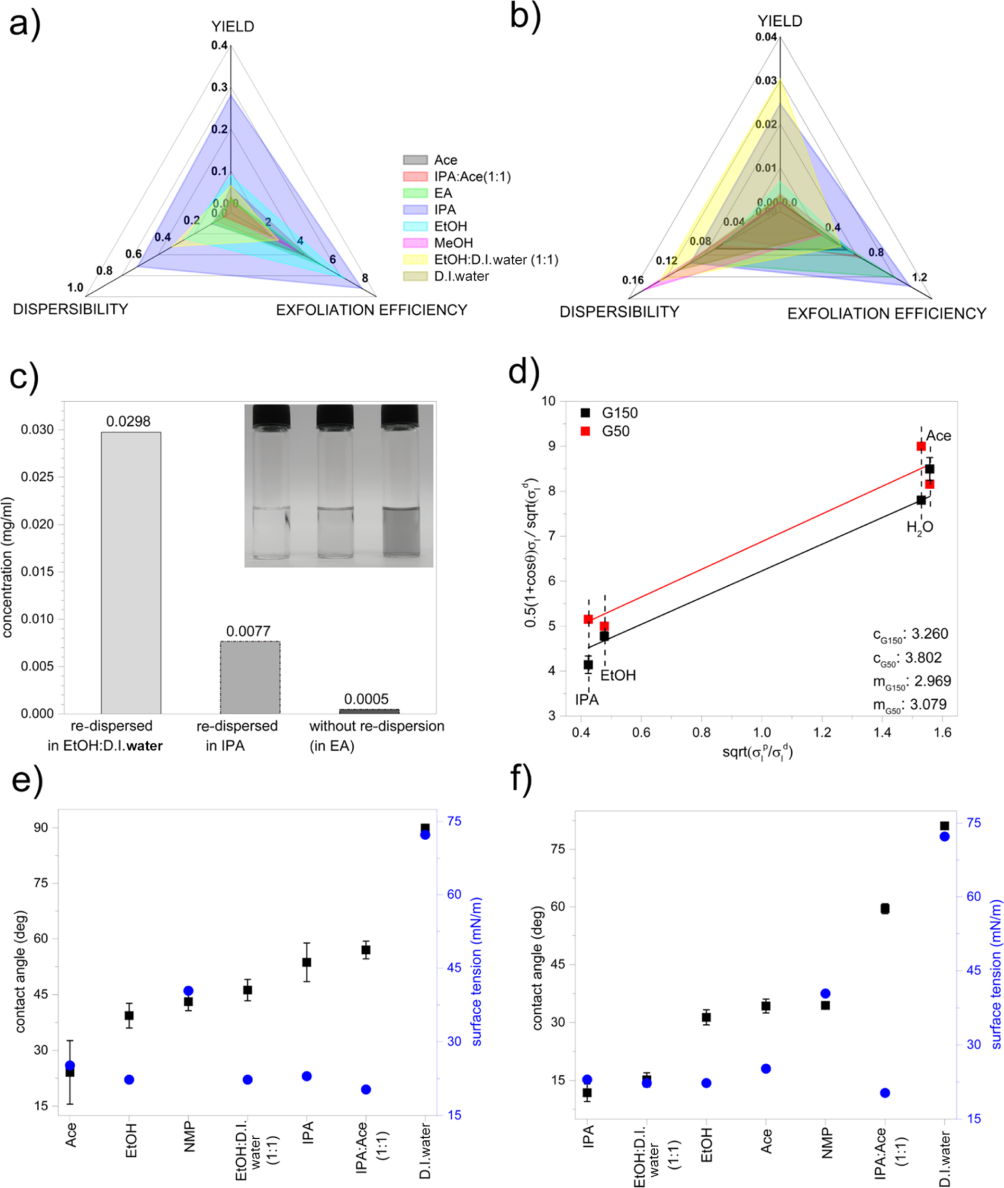
Yield of (a) GR150 and (b) GR50 in different solvent media, which is the combined contributions of solvent exfoliation efficiency and dispersibility. The graphene yield is determined through the conventional method. Exfoliation efficiency and graphene dispersibility are determined using the NMP-R and GS-R techniques, respectively. The unit of the axes is in mg/m^2^. (c) Concentration of GR50 exfoliated in EA as compared to its re-dispersion in IPA and EtOH/D.I. water (1:1). Inset: digital images of dispersions correspond to the *x*-axis arrangement. (d) Owens–Wendt–Rabel and Kaelble model plot for graphite surface energy determination. The interfacial contact angle measurement data evaluated by the Washburn method of the graphite starting materials G150 and G50 are shown in (e,f), respectively, and the exfoliation solvent media listed in the *x*-axis.

IPA has the highest dispersibility and exfoliation efficiency for GR150, that is, it produces the highest graphene yield (Figure 3a). For GR50, the yield is higher in EtOH/D.I. water than in IPA. This difference in yield is related to the significant contribution of graphene dispersibility in the process (Figure 3b). Also, the low GR50 yield in EA is mainly due to its low graphene dispersibility rather than the exfoliation efficiency (Figure 3b). To confirm the reliability of the GS-R and NMP-R approaches, we dispersed the dried product of SM-LPE in EA, a solvent of low GR50 dispersibility but high exfoliation efficiency, in the green solvents with high GR50 dispersibility, that is, IPA and EtOH/D.I. water (the UV–vis spectra are shown in S7). This resulted in the enhancement of GR50 yield by 16 times when re-dispersed in IPA (from 0.0005 to 0.0077 mg/mL) and 60 times (from 0.0005 to 0.0298 mg/mL) when re-dispersed in EtOH/D.I. water (Figure 3c), implying that the actual exfoliation efficiency of EA is higher than initially detected. All findings discussed above beg the question of what the reason behind the high exfoliation efficiency of green solvents (e.g., IPA) is. In order to understand the exfoliation efficiency, we studied the correlation between the graphite surface energy and solvent surface tension. We employed the Washburn method and evaluated the interfacial contact angle for G50 and G150 (S8.1), without the need of compressing it into pellets—required for a conventional contact angle approach. The properties (surface tension, density, and viscosity) of the test liquids used for the contact angle measurement are shown in Table S1. The contact angle obtained are used for the Owens–Wendt–Rabel and Kaelble (OWRK) plot (Figure 3d) for the graphite surface energy determination, calculated from the gradient and *y*-intercept of the plot. The solvents’ surface tension components data used for the surface energy calculation are listed in Table S2. Both G50 and G150 graphite exhibit different surface energies (S8.1). The interfacial contact angle between all tested solvents and G150 and G50 is lower than 90° (Figure 3e), confirming the feasibility of both graphite types to be wetted, as the lower interfacial contact angle between graphite and solvent contributes to the lower interfacial surface tension. Figure 3e,f shows, however, that not all solvents contribute to the wettability of the graphite materials in the same way, even those with a similar surface tension. For example, NMP, known as the best solvent for exfoliating and dispersing graphene, has poorer wettability than the number of green solvents. The calculated surface energies for G150 and G50 are 19.44 and 23.93 mN/m, respectively (Figure 3d), which are close to the surface tension for most tested green solvents except for that of D.I. water. NMP, however, has a much higher surface tension than the surface energies of both G150 and G50, and yet, it has a high exfoliation efficiency. Another possible way to explain this phenomenon is the concept of polar to dispersive component ratio of surface tension (σ_p_/σ_d_)^35,36^ The polar component, , and the dispersive component, , for graphite were calculated using the OWRK model (see S8). The analysis shows the difference in σ^p^/σ^d^ between G150 and G50 and the solvent [Δσ_(p/d)_] (Figure S7). Δσ_(p/d)_ for NMP is lower than that for green solvents while maintaining a high exfoliation efficiency, regardless of its large surface tension difference with G150 and G50 graphite. Similarly, IPA shows low Δσ(p/d), which explains its high exfoliation efficiency, close to that of NMP. On the contrary, Ace and D.I. water show high Δσ(p/d), which causes the low exfoliation efficiency.

**Figure 4:**
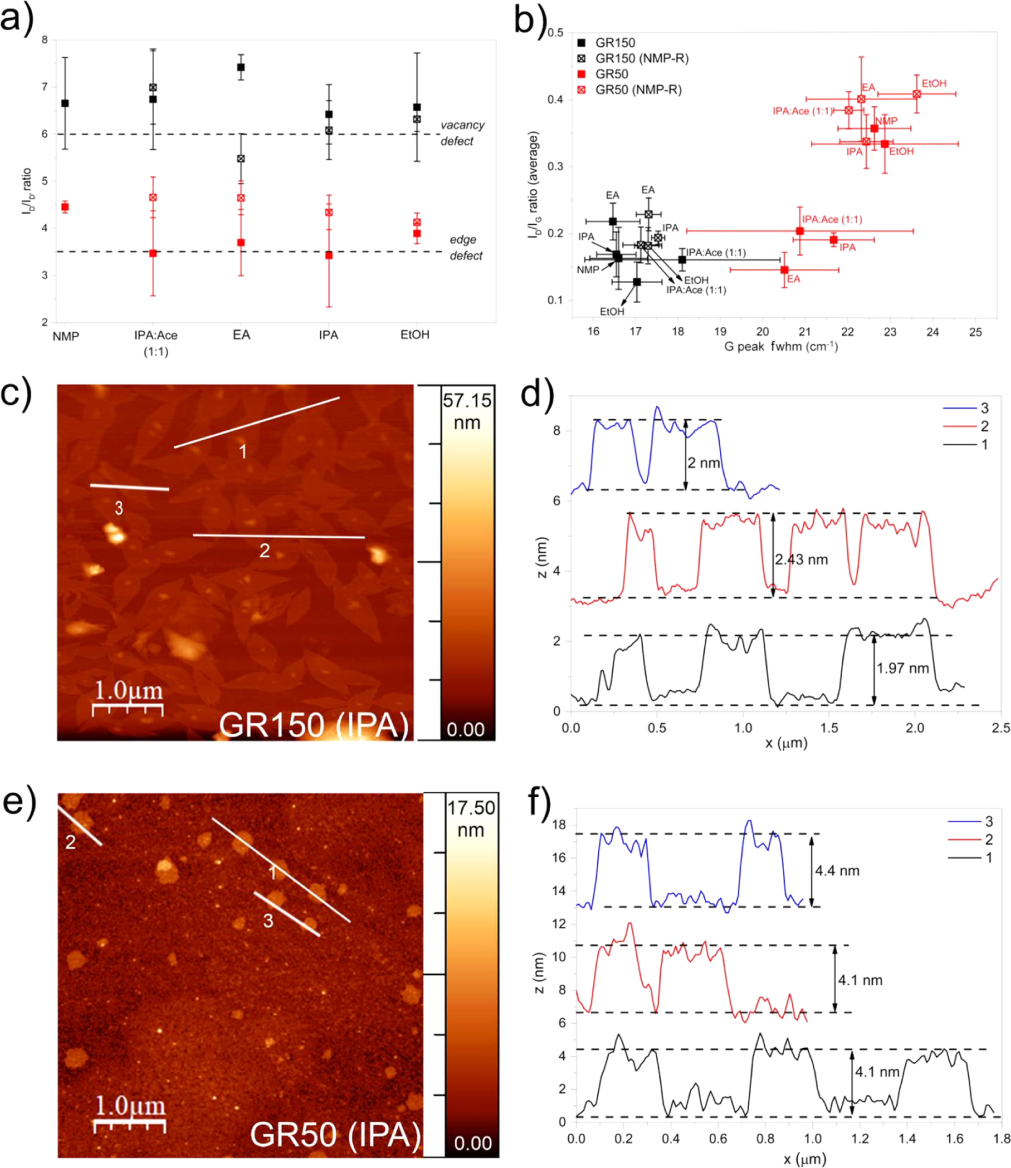
Raman spectroscopy analysis for GR150 and GR50 before and after NMP-R (a) on type of defects (D/D′ intensity ratio) for NMP, IPA/Ace (1:1), EA, IPA, and EtOH, (b) and defect density (D/G intensity ratio) against disorder (G peak fwhm). AFM thickness analysis performed on (c) GR 150 and (e) GR 50 exfoliated in IPA and displayed in (d,f), respectively.

To identify the type of defects and the quality of the exfoliated graphene, Raman spectroscopy analysis was employed (Figure S8). The evaluation of *I*_D_/*I*_D′_^37^ for all exfoliated graphene samples showed that the type of defects is similar to that of NMP-R graphene, except for that of the EA-exfoliated GR150 (Figure 4a). The variations of defects type and density/disorder across the samples (Figure 4a,b) are lower for both (with shorter error bars), NMP-R GR150 and GR50, when compared to those without re-dispersion. We analyzed the full width half maximum (fwhm) for the G peak in conjunction with the D to G intensity ratio (*I*_D_/*I*_G_) as it is confirmed to be a more accurate way to determine the lattice disorder within graphene.^38,39^ The G peak fwhm is higher for GR50 exfoliated in all investigated solvents; hence, GR50 has a higher lattice disorder than GR150 (Figure 4b). The NMP-R G50, however, is more disordered with a higher defect density. This implies that NMP not only improves the dispersibility and hence the yield of graphene but also more likely disperses the highly disordered graphene. The Raman spectra of GR150 exfoliated in IPA and NMP and the commercial graphene are shown in Figure S9. The spectra were normalized to the highest intensity G peak. We found no obvious difference neither in the defect density (D peak) nor the number of layers (2D peak). Atomic force microscopy (AFM) examination was used to determine the thickness of GR150 and G50 exfoliated in IPA. The exfoliated GR150 shows a very homogeneous leaf-like structure with a lateral size close to the micron scale and with the thickness ranging from 5 to 9 layers (Figure 4c,d). GR50 exfoliated in IPA, however, has a smaller lateral size but is thicker than the IPA-exfoliated GR150 (Figure 4e,f). This is strong evidence of how the structure and morphology of the initial graphite materials affect the quality and lateral size of the final product, and this must not be neglected by the graphene community.

## Conclusions

We showed direct evidence that the yield of graphene produced by LPE consists of two separate factors/components: (i) the solvent exfoliation efficiency and (ii) the solvent’s ability to disperse the exfoliated graphene. We systematically evaluated a wide range of green solvents and their mixtures together with the state-of-the-art NMP solvent, which is known as the best for LPE, as a control. Specifically, we studied the potential of NMP-R that we developed in assessing and quantifying the exfoliation efficiency of green solvents and the GS-R in assessing the graphene dispersibility of green solvents. From the study, the IPA solvent has a high exfoliation efficiency toward the exfoliation of GR150 graphene, which is comparable to that of NMP (despite its poor graphene dispersibility), and EtOH/D.I. water provides the highest GR50 yield mainly because of its good graphene dispersibility. We also verified the superior exfoliation efficiency of IPA by experimentally studying the surface energy and surface tension between graphite and the solvent using the Washburn method. By re-dispersing the graphene exfoliated in the solvent with a high exfoliation efficiency but poor dispersibility into a solvent with a relatively high dispersibility, the graphene concentration can be improved up to 16 times. Our NMP-R and GS-R approaches can be applied to LPE on any type of layered material in order to evaluate the potential of green solvents for obtaining the highest possible yield of the material.

## Supporting Information Available

Experimental methods, graphite starting materials’ characterization, UV–vis spectra, contact angle and surface energy measurements (Washburn method), and Raman spectra (PDF)

## Abbreviations

Ace: acetone
AFM: atomic force microscopy
BET: Brunauer–Emmet–Teller
D.I. water: deionized water
DMF: dimethylformamide
EtOH: ethanol
EA: ethyl acetate
fwhm: full width at half-maximum
GS-LPE: green solvent-liquid phase exfoliation
GS-R: green solvent-re-dispersion
IPA: isopropanol
LPE: liquid phase exfoliation
MeOH: methanol
NMP: 1-methyl-2-pyrrolidone
NMP-R: NMP-re-dispersion
OWRK: Owens–Wendt–Rabel and Kaelble
SM: shear mixing
SM-LPE: shear mixing-liquid phase exfoliation
US: ultrasonication
