# Supplementary material for: Direct Evidence of the Exfoliation Efficiency and Graphene Dispersibility of Green Solvents toward Sustainable Graphene Production
Source: ACS Sustain Chem Eng. 2022 Dec 9;11(1):58–66. doi: 10.1021/acssuschemeng.2c03594 (PMC9832534; doi:10.1021/acssuschemeng.2c03594)
Supplement: Supplementary file 1 — sc2c03594_si_001.pdf [file sc2c03594_si_001.pdf]

Supporting Information

Direct evidence of exfoliation efficiency and graphene dispersibility of green solvents towards sustainable graphene production

Kai Ling Ng‡[1], Barbara M Maciejewska‡[1], Ling Qin[2], Colin Johnston[1], Jesus Barrio[3], Maria-Magdalena Titirici[3], Iakovos Tzanakis[4], Dmitry G Eskin[5], Kyriakos Porfyrakis[6], Jiawei Mi[2], Nicole Grobert*[1,7]

[1] *Department of Materials, University of Oxford, Parks Road, Oxford, OX1 3 PH, UK*
[2] *Department of Engineering, University of Hull, Cottingham Rd, Hull, HU6 7RX, UK*
[3] *Department of Chemical Engineering, Imperial College London, South Kensington Campus, London, SW7 2AZ, UK*
[4] *School of Engineering, Computing and Mathematics, Oxford Brookes University., College Cl, Wheatley, Oxford, OX33 1HX, UK*
[5] *Brunel Centre for Advanced Solidification Technology, Brunel University London, Kingston Lane, UB8 3PH, UK*
[6] *Faculty of Engineering and Science, University of Greenwich, Central Avenue, Chatham Maritime, Kent, ME4 4TB, UK*
[7] *Williams Advanced Engineering, Grove, Oxfordshire, OX12 0DQ, UK*

***Corresponding author**

Nicole Grobert

nicole.grobert@materials.ox.ac.uk

Number of pages: 14

Number of figures: 9

Number of tables: 2

Table of Contents:

1 Materials and experimental methods **2**
1.1 Materials 2
1.2 Experimental methods 2
1.2.1 Shear mixing exfoliation 2
1.2.2 NMP-Redispersion and Green Solvent-Redispersion 2
2 Characterisation of graphite and graphene **3**
2.1 Scanning Electron Microscopy 3
2.2 Brunauer Emmet Teller 3
2.3 Raman spectroscopy 3
2.4 UV-Vis spectroscopy 3
2.5 Atomic Force Microscopy (AFM) 3
3 Graphite characterisation **4**
4 UV-Vis calibration plots **5**
5 UV-Vis concentration analysis on graphene produced **6**
5.1 Graphene concentrations without redispersion 6
5.2 Graphene concentrations after redispersion in NMP or green solvent 6
6 UV-Vis Spectrometry of GR150 and GR50 before and after GS-R **7**
7 UV-Vis spectroscopy of EA-exfoliated GR50 dispersed in IPA and EtOH:D.I. **8**
8 Interfacial contact angle measurement (Washburn method) and surface energy analysis **8**
8.1 Graphite surface energy calculation 9
8.2 Exfoliation efficiency and surface tension component ratio *(σ_p / σ_d)* analysis 10
9 Raman spectroscopy of graphene with and without NMP-R **11**
10 Raman spectroscopy of IPA- and NMP-exfoliated graphene in comparison to the commercial graphene **12**
References: **13**

1 Materials and experimental methods

1.1 Materials

Sigma Aldrich 100-mesh graphite, Alfa Aesar 300-mesh graphite, 2-propanol (Sigma Aldrich, ≥ 99.8 %, GC grade), Acetone (Sigma Aldrich, ≥ 99.5 %, GC grade), Ethanol (Sigma Aldrich, ≥ 99.8 %, GC grade), Ethyl Acetate (Sigma Aldrich, ≥ 99.5 %, GC grade), Methanol (Sigma Aldrich, ≥ 99.9 %, HPLC grade), 1-Methyl-2-Pyrrolidinone (Sigma Aldrich, ≥ 99.5 %, anhydrous)

1.2 Experimental methods

1.2.1 Shear mixing exfoliation

Sigma-Aldrich graphite (SA, 100 mesh) and Alfa-Aesar graphite (AA, 300 mesh) were exfoliated with a concentration of 25 mg/mL in a range of non-toxic, low boiling point solvents (D.I., EtOH, MeOH, EA, Ace, IPA) and solvent mixtures [(EtOH:D.I. (1:1 vol. %), IPA:Ace (1:1 vol. %)] using a shear mixer at 5000 rpm for 3 hours. The total volume of 150 ml was kept constant during shear mixing by 'topping up' with the relevant solvent. After exfoliation, the samples were centrifuged at 500 rpm for 1 hour to separate the exfoliated graphene from large graphite aggregates for characterisation of the supernatant.

1.2.2 NMP-Redispersion and Green Solvent-Redispersion

NMP-Redispersion: All samples exfoliated in green solvents (see list in **1.2.1**) were dried at 100 ^{o}C in a vacuum oven to evaporate the solvent after exfoliation. NMP (equal volume as the evaporated amount) was added**.**

GS-Redispersion: All samples exfoliated in NMP were washed with ethanol using vacuum filtration with 0.22 micron PTFE membrane before drying them in a vacuum oven at 100 ^{o}C to evaporate the remaining solvent. Same volume of green solvents were added to disperse the NMP exfoliation products. For both NMP and GS-R, a vortex mixer was used to homogenise the samples to eliminate possible effects of ultrasonic cavitation. After homogenisation, the samples were centrifuged at 500 rpm for 1 hour and the supernatant was taken for concentration analysis. (*Exfoliation products are the graphene-containing products with graphite aggregates obtained after exfoliation and before centrifugation.)

2 Characterisation of graphite and graphene

The samples were characterised by means of scanning electron microscopy (SEM), Brunauer-Emmet-Teller (BET), Raman spectroscopy, UV-vis spectroscopy, and atomic force microscopy. Sample preparation and operating conditions are described below:

2.1 Scanning Electron Microscopy

The graphite was sparingly sprinkled onto sticky copper tape for SEM analysis of the raw materials. SEM micrographs were taken at 5 kV, using JEOL JSM-840F with cold cathode field emission gun.

2.2 Brunauer Emmet Teller

Brunauer Emmet Teller (BET) measurements were performed under nitrogen atmosphere (nitrogen adsorption-desorption isotherm at 77K) using a Micromeritics' Gemini Vll . The samples were degassed at 225 ˚C overnight under nitrogen prior to the measurement. Sample mass of 0.3228 g was used on all the samples.

2.3 Raman spectroscopy

The graphene dispersions were dropcast onto a Si/SiO_2 wafer and dried under ambient air for Raman spectroscopy. For the graphene samples dispersed in NMP, the graphene dispersion was dropcast onto a Si/SiO_2 wafer, then dried at 150 ^oC on a hotplate. The Raman D, D' and G peaks were fitted using a Lorentzian function. Raman Spectroscopy was performed using the Horiba Scientific LabRAM ARAMIS. An objective lens of x50, laser wavelength of 532 nm and laser intensity of 10 % were used to acquire the Raman spectra.

2.4 UV-Vis spectroscopy

The UV-Vis spectra were acquired using Varian Cary 5000. The concentration of graphene dispersion was calculated using the Lambert-Beer Law $A = \varepsilon l c$ **(Eq. 1)** A absorbance, ε is the molar absorptivity [ml/mg · m], l is the optical path length (0.01 m) and c is the concentration (mg/ml) of the attenuating species (graphene). The molar absorptivity, was determined using the calibration curves (see **4**).

2.5 Atomic Force Microscopy (AFM)

An Agilent 5400 AFM was used to evaluate the sample morphology and graphene flake thickness. The samples were prepared by drop casting graphene solutions on a Si/SiO_2 wafer placed on a hot plate, heated to 80 ^oC (for green solvents) and to 150 ^oC (for NMP).

3 Graphite characterisation

Two different commercial graphite sources (See **1.1**) were used for exfoliation and characterised by scanning electron microscopy (SEM) and Raman.

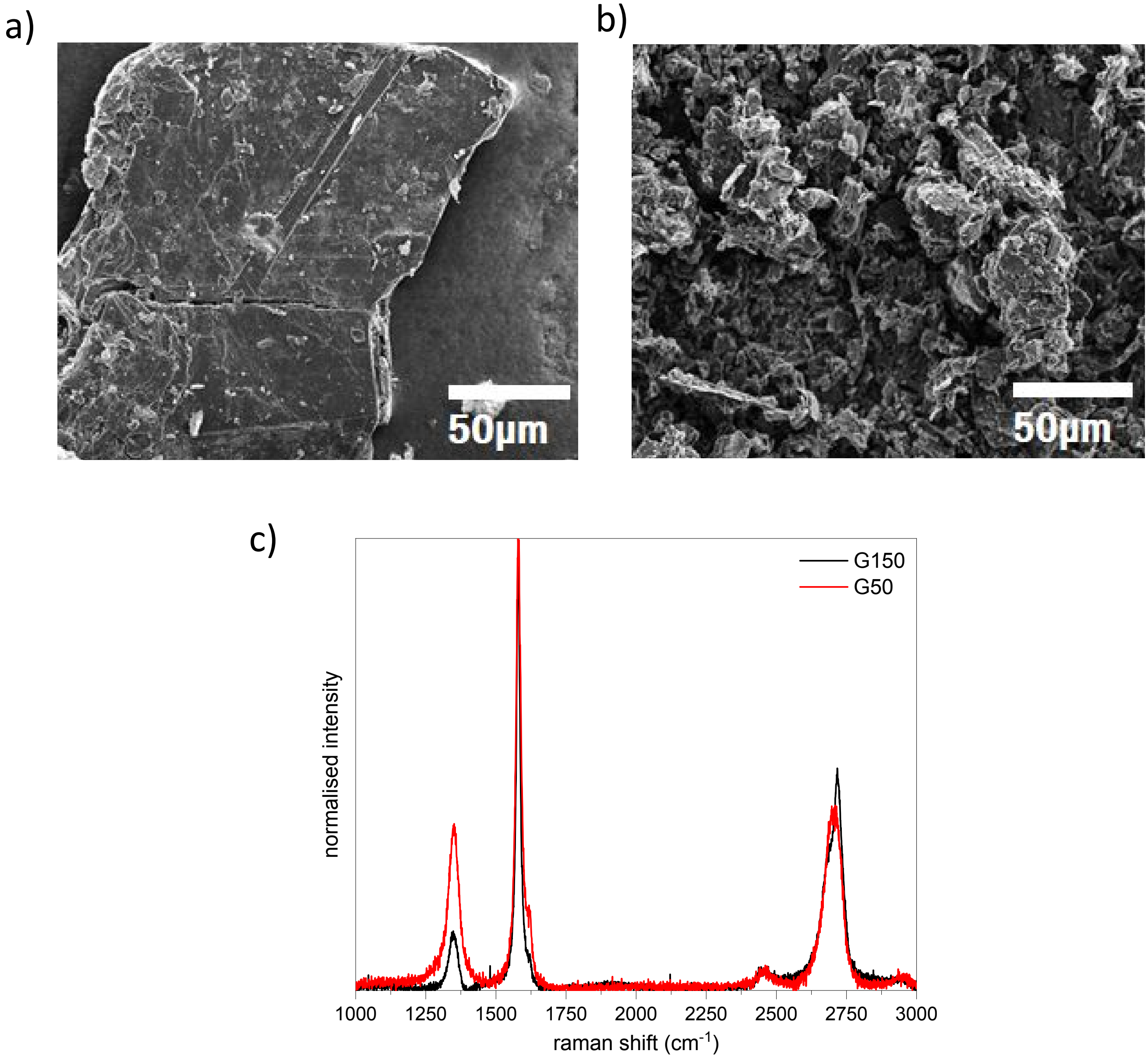

Fig. S1: *SEM images of commercial graphite (a) G150 and (b) G50; (c) Raman spectra of G150 and G50*

4 UV-Vis calibration plots

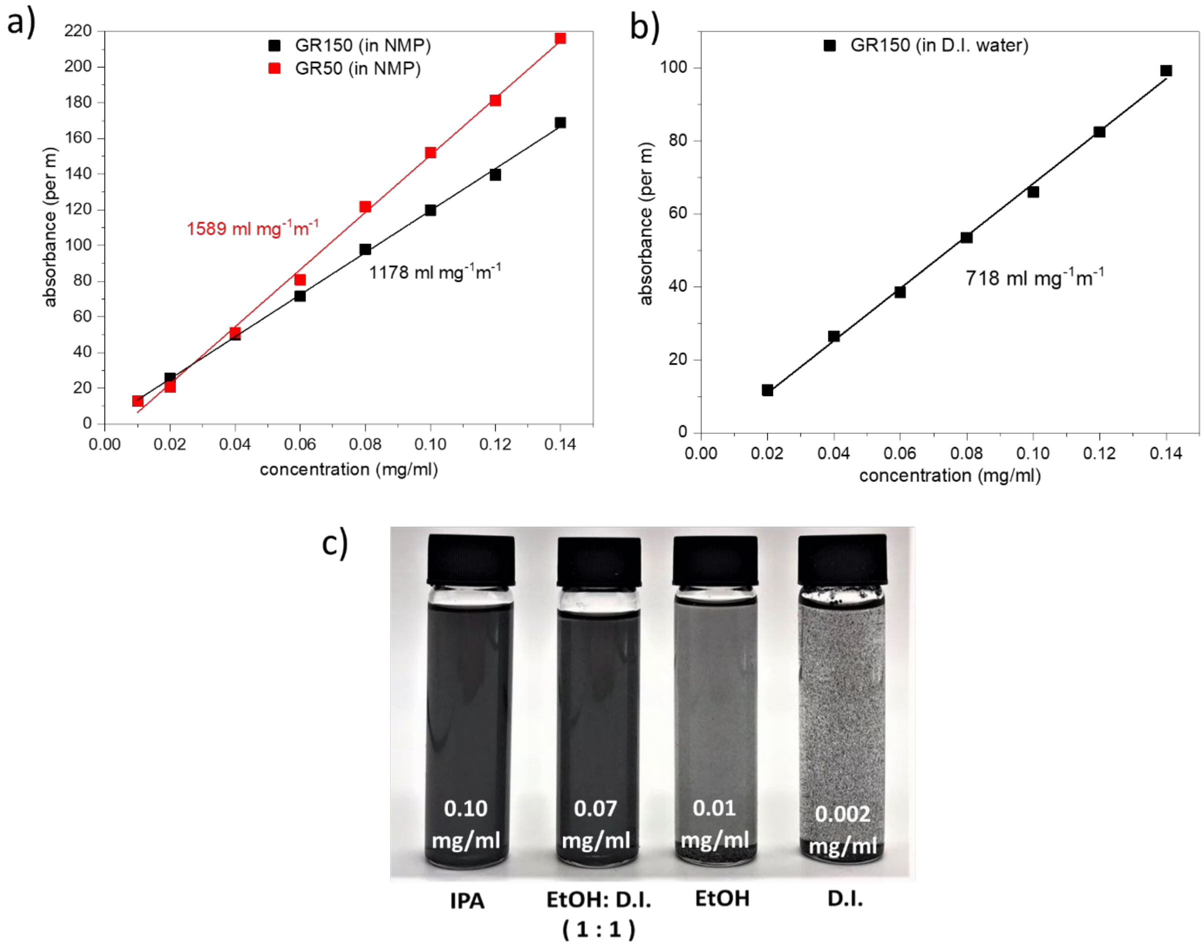

Fig. S2: *UV-Vis calibration curves for GR150 and GR50 in a) NMP; b) GR150 in D.I.; c) 0.1 mg/ml GR150 dispersed in different solvents. UV-Vis spectroscopy, using the absorptivity value of 1178 ml/mg/m revealed the following graphene concentrations 0.1 mg/ml for IPA, 0.07 mg/ml for EtOH:D.I., 0.01 mg/ml for EtOH, and 0.002mg/ml for D.I.*

Absorptivity values obtained for graphene dispersed in NMP (**Figure S2a**) and D.I. water (**Figure S2b**). The lower the absorptivity value, the higher the calculated graphene concentration.

Figure S2c illustrates dispersed graphene (of equal mass) in solvents with different dispersibility. It is visible that graphene is less well dispersed in EtOH and D.I.; *i.e.*, their ability to disperse graphene is lower which leads to low absorbance and thus lower concentration for a given mass of graphene.

5 UV-Vis concentration analysis on graphene produced

Figures S3-S4 show the concentration of GR150 and GR50 (produced from the same mass of G150 and G50 respectively) in different solvents.

5.1 Graphene concentrations without redispersion

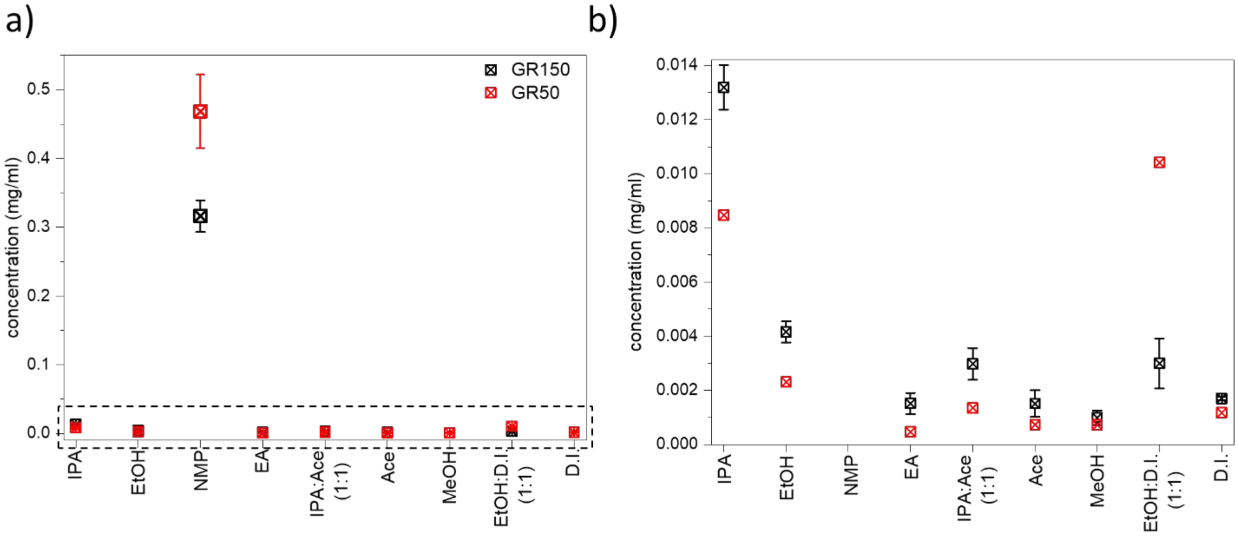

Fig. S3: *(a) and (b) revealing the concentrations of as produced GR150 and GR50 in different solvents. (b) 'Close-up' of (a) to reveal the differences between the solvents with NMP off scale.*

5.2 Graphene concentrations after redispersion in NMP or green solvent

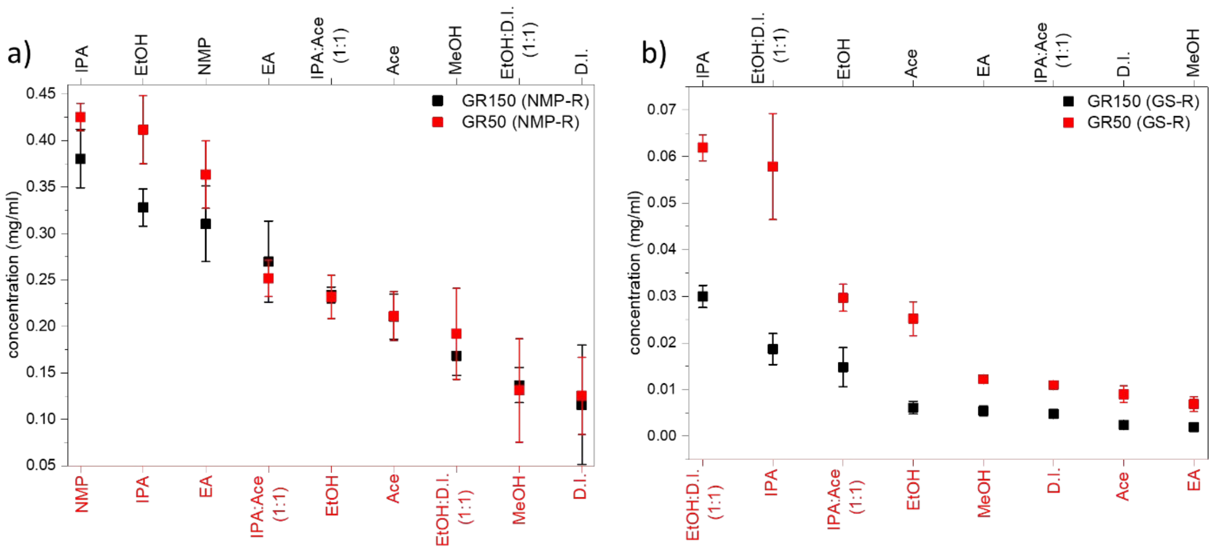

Fig. S4: *Concentration of GR150 and GR50 after redispersion in (a) NMP. The green solvents used for the exfoliation (x-axis) are arranged in decreasing order of concentration/exfoliation efficiency. (b) Concentration of GR150 and GR50 after redispersion in green solvents. The green solvents (x-axis) are arranged in the decreasing order of concentration/dispersibility.*

6 UV-Vis Spectrometry of GR150 and GR50 before and after GS-R

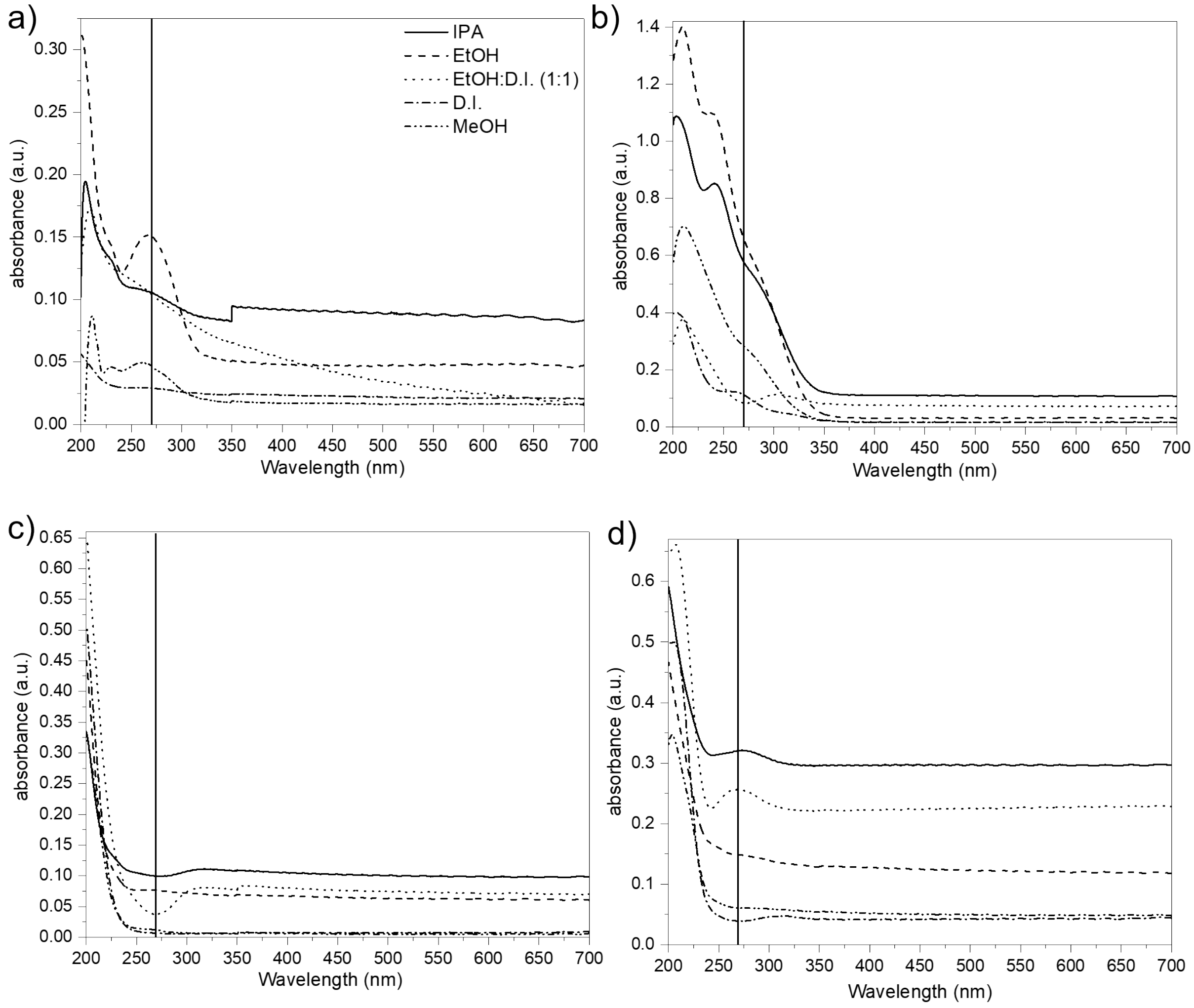

Fig. S5: *UV- Vis spectra of (a) GR150, (b) GR50, (c) GR150 (GS-R) and (d) GR50 (GS-R). The shift in pi-pi* absorption peak position from 270 nm (indicated by the vertical lines in the graphs) is the typical absorption wavelength for pristine graphene and is depending on the dispersing solvent medium used.*

7 UV-Vis spectroscopy of EA-exfoliated GR50 dispersed in IPA and EtOH:D.I.

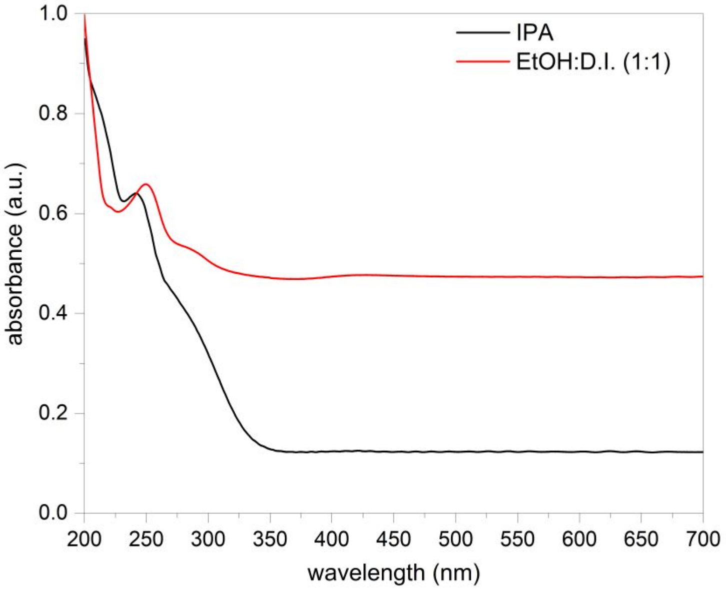

Fig. S6: *UV-Vis spectra of EA exfoliated GR50 redispersed in IPA (black) and EtOH:D.I. (1:1) (red).*

8 Interfacial contact angle measurement (Washburn method) and surface energy analysis

The interfacial contact angle (θ) measurement for graphite flakes (G150) and graphite powder (G50) in selected test liquids (exfoliation solvents used in this work) was done by means of KRUSS Tensiometer K100. Hexane was used to determine the capillary constant. The contact angle was calculated using the following equation:

$$\frac{m^2}{t} = \frac{c \cdot \rho^2 \cdot \sigma \cdot \cos\theta}{\eta} \qquad \textbf{(eq. 2)}$$

m [g] mass of the tube and sample; t [s] flow time; c [mm^5] capillary constant of the sample (c_{G150} = 2.76847335 mm^5; c_{G50} = 0.6138338 mm^5; ρ (g/ml) density; σ (mN/m) surface tension; η (mPa · s) viscosity; and θ (deg) contact angle of the test liquid.

The viscosity, surface tension, and density were obtained from KRUSS instrument database (Table S1) apart from the solvent mixtures of EtOH:D.I (1:1) and IPA:Ace (1:1) which were taken from the literature as referenced:

Table S1 Surface tension viscosity and density values for used test liquids.

Solvents (Test Liquid)	Surface Tension [mN/m]	Density [g/ml]	Viscosity [mPa · s]
IPA	23.0	0.7860	1.960
EtOH:D.I. (1:1)	22.3 [1]	0.7895 [2]	1.341 [3]
EtOH	22.3	0.7858	1.100
Ace	25.2	0.7910	0.295
NMP	40.4	1.0280	1.661
IPA:Ace (1:1)	20.3 [4]	0.7808 [5]	0.308 [6]
D.I.	72.3	0.9970	0.900

8.1 Graphite surface energy calculation

The surface energies of G150 and G50 graphite were calculated using Owens-Wendt-Rabel & Kaelble model (OWRK) equation:

$$\frac{\sigma_l(\cos\theta+1)}{2(\sqrt{\sigma_l^d})} = (\sqrt{\sigma_s^p})\frac{\sqrt{\sigma_l^p}}{\sqrt{\sigma_l^d}} + \sqrt{\sigma_s^d} \qquad \textbf{(eq.3)}$$

σ_l [mN/m] overall surface tension of liquid/solvent; σ_s^d dispersive component of the graphite surface energy; σ_l^d surface tension of liquid/solvent; σ_s^p polar component of graphite surface energy; σ_l^d liquid surface tension; θ (deg) interfacial contact angle between graphite and liquid

By plotting the linear graph of

$$\frac{\sigma_l(\cos\theta+1)}{2(\sqrt{\sigma_l^d})} \quad \text{against} \quad \frac{\sqrt{\sigma_l^p}}{\sqrt{\sigma_l^d}}$$

as shown in Fig. S8, the overall surface energy of graphite, σ_s can be estimated from the slope and y-intercept of the graph, which gives the value for

$$\sqrt{\sigma_s^p} \text{ and } \sqrt{\sigma_s^d} \text{ respectively.}$$

The equation used for calculating the surface energy is:

$$\sigma_s = \sigma_s^p + \sigma_s^d \qquad \textbf{(eq.4)}$$

The overall surface energy of both G150 and G50 graphite (σ_{G150} and σ_{G50}) were calculated as follows:

$$\sigma_{G150} = \sigma_{G150}^p + \sigma_{G150}^d = \left(\sqrt{\sigma_{G150}^p}\right)^2 + \left(\sqrt{\sigma_{G150}^d}\right)^2$$

$$= (2.969)^2 + (3.260)^2 = \textbf{19.441 mN/m}$$

$$\sigma_{G50} = \sigma_{G50}^p + \sigma_{G50}^d = \left(\sqrt{\sigma_{G50}^p}\right)^2 + \left(\sqrt{\sigma_{G50}^d}\right)^2$$

$$= (3.079)^2 + (3.802)^2 = \textbf{23.930 mN/m}$$

The values used for polar and dispersive components of solvent surface tension for graphite surface energy calculation are listed as follows:

Table S2 *The polar and dispersive surface tension component estimated for the test liquids.*

Solvents	Polar Surface Tension Component, σ_l^p [mN/m]	Dispersive Surface Tension Component, σ_l^d [mN/m]
EtOH	4.4	19.3
D.I.	51	21.8
Ace	16.5	6.8
NMP	11.58	29.21
IPA	3.5	19.5

8.2 Exfoliation efficiency and surface tension component ratio *(σ_p / σ_d)* analysis

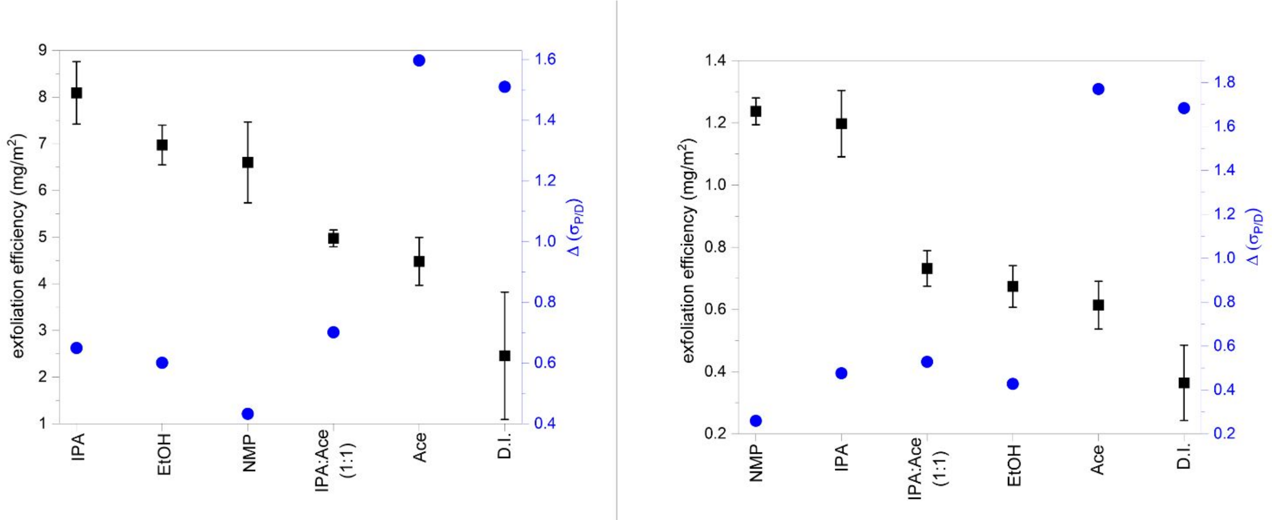

Fig. S7: *Exfoliation efficiency of a) G150 graphite and b) G50 graphite, and it's relationship with the polar to dispersive ratio of surface tension difference (Δσ(p/d)) between solvents and graphite. Low (Δσ(p/d)) gives higher exfoliation efficiency.*

9 Raman spectroscopy of graphene with and without NMP-R

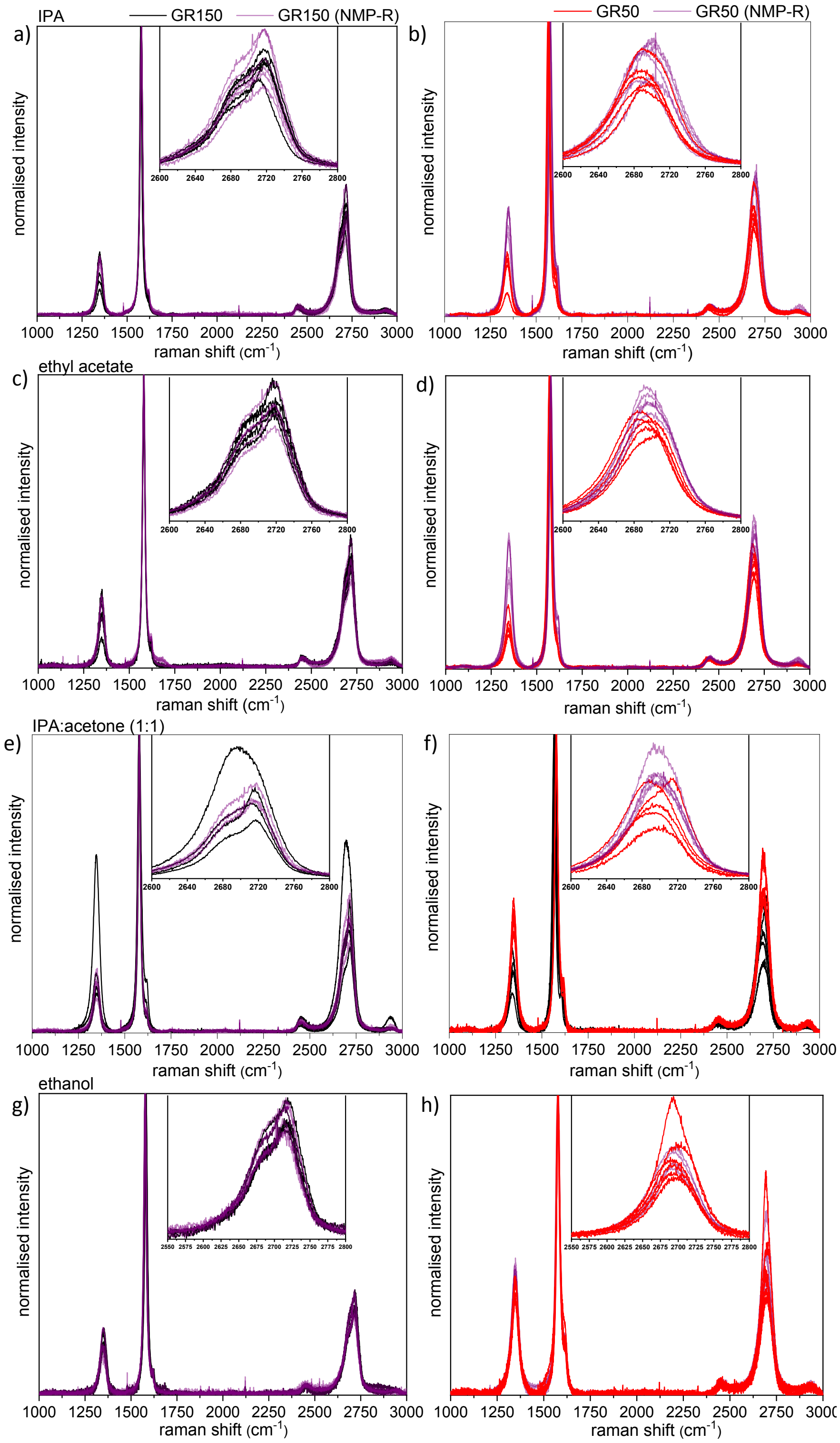

Fig. S8: *Comparison of GR150 and GR50 Raman spectra with (GR150 black, GR50 red) and without (purple) NMP-R. The Raman spectra were normalised against the G peak. The 2D peaks are shown in the inset of each graph; a) GR150 exfoliated in IPA, b) GR50 exfoliated in IPA, c) GR150 exfoliated in ethyl acetate, d) GR50 exfoliated in ethyl acetate, e) GR150 exfoliated in IPA:acetone (1:1). f) GR50 exfoliated in IPA:acetone, g) GR150 exfoliated in ethanol and h) GR50 exfoliated in ethanol.*

10 Raman spectroscopy of IPA- and NMP-exfoliated graphene in comparison to the commercial graphene

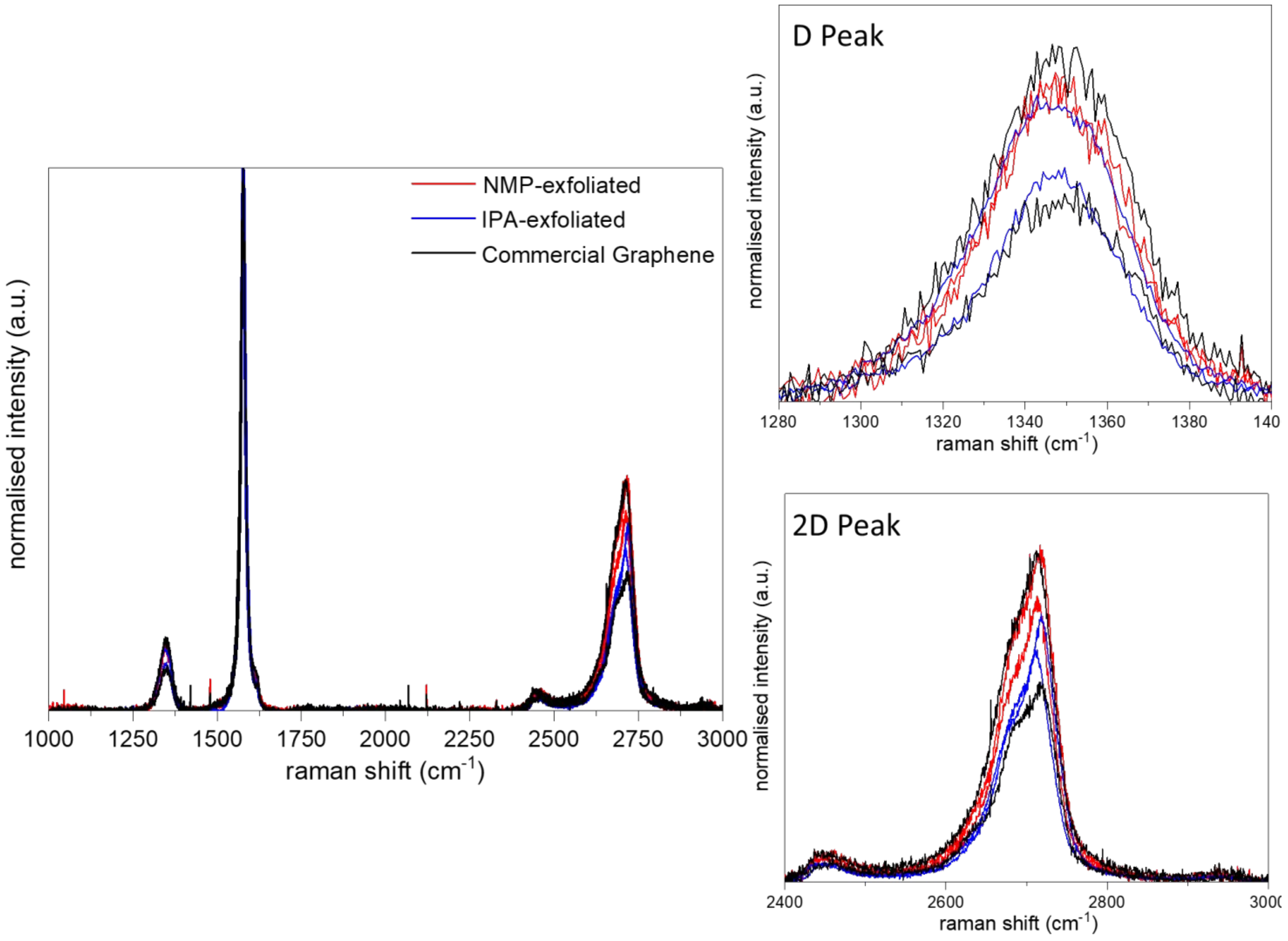

Fig. S9: *Comparison of IPA- and NMP-exfoliated GR150 Raman spectra with commercial graphene. Two sepctra are collected for each sample. The Raman spectra are normalised to the G peak. D and 2D peaks give information on the defect and layer number. There is no obvious difference in the D and 2D peaks between commercial, IPA- and NMP-exfoliated graphene. The type of exfoliation solvents used has no significant effect on the graphene quality.*

References:

1. **Ethanol-Water Mixture Surface Tension**, Datasheet from "Dortmund Data Bank (DDB) – Thermophysical Properties Edition 2014" in SpringerMaterials (https://materials.springer.com/thermophysical/docs/msft_c11c174), J. Gmehling, Editor., Springer-Verlag Berlin Heidelberg & DDBST GmbH, Oldenburg, Germany.

2. **Ethanol-Water Density**, Datasheet from "Dortmund Data Bank (DDB) – Thermophysical Properties Edition 2014" in SpringerMaterials (https://materials.springer.com/thermophysical/docs/ve1_c11c174), J. Gmehling, Editor., Springer-Verlag Berlin Heidelberg & DDBST GmbH, Oldenburg, Germany.

3. **Ethanol-Water Mixture Viscosity**, Datasheet from "Dortmund Data Bank (DDB) – Thermophysical Properties Edition 2014" in SpringerMaterials (https://materials.springer.com/thermophysical/docs/vism_c11c174), J. Gmehling, Editor., Springer-Verlag Berlin Heidelberg & DDBST GmbH, Oldenburg, Germany.

4. **Binary Mixtures**, Wohlfarth, C. and B. Wohlfarth, Datasheet from Landolt-Börnstein - Group IV Physical Chemistry · Volume 16: "Surface Tension of Pure Liquids and Binary Liquid Mixtures" in SpringerMaterials (https://doi.org/10.1007/10560191_4), M.D. Lechner, Editor., Springer-Verlag Berlin Heidelberg.

5. **Acetone-2-Propanol Density**: Datasheet from "Dortmund Data Bank (DDB) – Thermophysical Properties Edition 2014" in SpringerMaterials (https://materials.springer.com/thermophysical/docs/ve1_c4c95), J. Gmehling, Editor., Springer-Verlag Berlin Heidelberg & DDBST GmbH, Oldenburg, Germany.

6. **Acetone-2-Propanol Mixture Viscosity**: Datasheet from "Dortmund Data Bank (DDB) – Thermophysical Properties Edition 2014" in SpringerMaterials (https://materials.springer.com/thermophysical/docs/vism_c4c95), J. Gmehling, Editor., Springer-Verlag Berlin Heidelberg & DDBST GmbH, Oldenburg, Germany.

7. **Exfoliation of WS2 in the semiconducting phase using a group of lithium halides: A new method of Li intercalation**, A Ghorai, A Midya, R Maiti, SK Ray, *Dalt. Trans.* (2016). doi:10.1039/c6dt02823c

8. **Surface Tension Components Based Selection of Cosolvents for Efficient Liquid Phase Exfoliation of 2D Materials**. J Shen *et al.*, Small (2016). doi:10.1002/smll.201503834

9. **Liquid Phase Exfoliation of Two-Dimensional Materials by Directly Probing and Matching Surface Tension Components**. J Shen *et al.*, Nano Lett. (2015). doi:10.1021/acs.nanolett.5b01842
